# Protocol for generating liver metastasis microtissues to decipher cellular interactions between metastatic intestinal cancer and liver tissue

**DOI:** 10.1016/j.xpro.2024.103575

**Published:** 2025-01-20

**Authors:** Maria Lamprou, Ana Krotenberg Garcia, Saskia Jacoba Elisabeth Suijkerbuijk

**Affiliations:** 1 Division of Developmental Biology, Institute of Biodynamics and Biocomplexity, Department of Biology, Faculty of Science, Utrecht University, Padualaan 8, Utrecht 3584 CH, the Netherlands

**Keywords:** Cancer, Microscopy, Organoids

## Abstract

Cell competition is a quality control mechanism that promotes elimination of suboptimal cells relative to fitter neighbors. Cancer cells exploit these mechanisms for expansion, but the underlying molecular pathways remain elusive. Here, we present a protocol for generating matrix-free microtissues recapitulating cellular interactions between intestinal cancer and hepatocyte-like cells using microscopy or transcriptomics/proteomics. We describe steps for generating and differentiating liver progenitor organoids and microtissue formation. We then detail procedures for immunofluorescence staining, mounting microtissues, and sorting cells.

For complete details on the use and execution of this protocol, please refer to Krotenberg Garcia et al.^1^

## Highlights

• Technique to generate mixed microtissues
• Follow complex cellular interactions during metastasis to the liver
• Analysis of cell competition in space and time
• Adaptable to other tumor types that are prone to metastasize to the liver

## Abstract (editor highlights)

Publisher’s note: Undertaking any experimental protocol requires adherence to local institutional guidelines for laboratory safety and ethics.

## Graphical abstract

**Figure:**
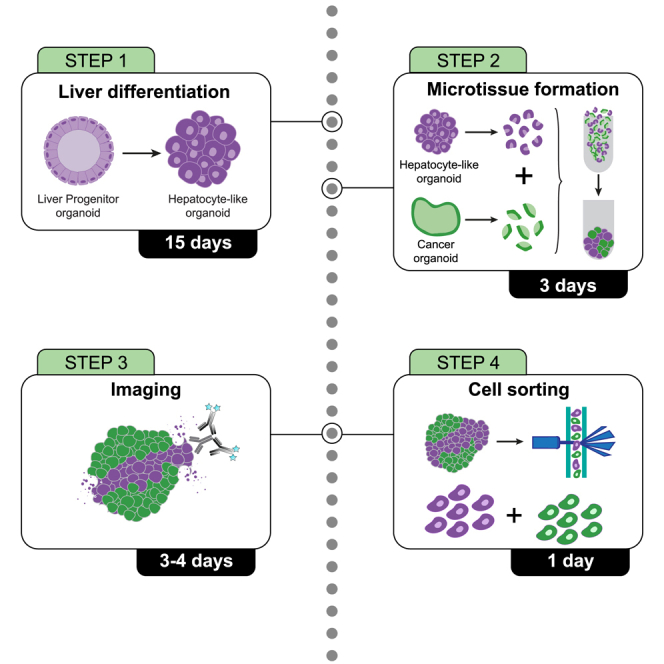

## Before you begin

Cell competition acts as an intercellular quality control mechanism that preserves tissue integrity by eliminating cells with lower fitness.^2^^,^^3^ Our recent findings show a crucial role of cell competition in intestinal cancer metastasis to the liver. Interestingly, utilizing a novel microtissue model, we outlined a stepwise process by which metastatic intestinal cancer cells exploit healthy liver cells as scaffold for expansion at the metastatic site. In turn cancer proliferation subsequently induces apoptosis-dependent elimination of hepatocytes via mechanisms that yet remain elusive. This highlights how microtissues can be used to unravel the impact of intercellular interactions on metastatic outgrowth.^1^

### Methodology and considerations

Here, we present a detailed protocol for generating matrix-free mixed microtissues as a novel three-dimensional (3D) model to investigate competitive interactions during intestinal cancer liver metastasis. This model was based on microtissues developed from various organs, such as the brain, heart and liver, which have significantly advanced our understanding of how their respective tissues respond to different types of damage or drug treatments.^4^^,^^5^^,^^6^^,^^7^^,^^8^^,^^9^ To generate liver metastasis microtissues, we employ intestinal *Apc*^−/−^*Kras*^G12D/WT^*Trp53*^-/R172H^ cancer and hepatocyte-like organoid cultures.^1^^,^^10^ Due to their structure, microtissues preserve cell differentiation and provide a robust and physiologically relevant platform for investigating mechanisms of intercellular communication.

For experiments based on this protocol, it is highly recommended to label each individual cell population. In the following sections, we provide guidelines for forming microtissues using membrane-bound tdTomato-labeled wild-type hepatocyte-like cells derived from *in vitro* differentiation of murine liver progenitor organoids^1^^,^^11^^,^^12^ and small intestinal cancer cells from *Villin-CreER*^*T2*^ *Apc*^*fl/fl*^ *Kras*^*G12D/WT*^ *Trp53*^*fl/R172H*^ transgenic mice^10^^,^^13^ labeled with Dendra2 (introduced via lentiviral transduction^14^). Murine adult-stem cell derived organoids were chosen due to the availability of in-house transgenic mouse models. Importantly, this enabled comparison of competitive interactions occurring during primary intestinal cancer growth and metastasis.^1^^,^^9^ While generation of microtissues from iPSC- and/or human-derived organoid lines is expected to be feasible, this has not been experimentally explored and the optimal culture conditions should first be determined.

Several considerations should be made when determining the optimal ratio of mixing two cell populations for microtissue formation: 1) the intrinsic physiological features of the cell populations, such as proliferative potential, 2) the stage of metastatic growth to be investigated (e.g., seeding or micro-metastasis formation) and 3) the type of cellular interactions (e.g., short- vs. long-ranged). For instance, in our model that represents liver tissue replacement during macro-metastasis outgrowth of highly proliferative intestinal cancer cells, a 2:1 ratio of wild-type hepatocyte-like to small intestinal cancer cells was found to be optimal. This ratio prevented biased out-competition caused by insufficient material of either cell populations, recapitulated advanced metastatic outgrowth and provided sufficient interaction surface for both populations.

### Applications

This protocol has wide-ranging applications and, when combined with microscopy or flow cytometry, can be employed to examine cellular interactions both at the single-cell level and across entire populations. Additionally, chemical or genetic manipulation along with downstream analyses like (single cell) mRNA sequencing or proteomics, can reveal specific molecular pathways. Furthermore, liver is a frequent metastatic site for tumors originating from the gastrointestinal tract, pancreas, breast, and lung and all of them exhibit common distinct histopathological growth patterns. The described liver metastasis microtissues are particularly applicable to investigate metastasis of replacement growth pattern, characterized by low stromal and immune cell infiltration, direct healthy-cancer cell interactions and poor patient prognosis.^15^ Therefore, microtissues are a promising model for investigating competitive interactions in liver metastasis not only from intestinal cancer but also from other primary tumors, such as those originating from the breast and pancreas.

### Material and reagent preparation

**Timing:1day**

1. Thaw Reduced Growth Factor Basement Membrane Extract Type 2 (BME2) on ice or at 4°C (8–16 h).
2. Ensure availability of 12-well untreated polystyrene plates for organoid culture and round (U-bottom) plates for microtissue formation.
3. Prepare all buffers and media according to the instructions in the “materials and equipment” section and store them appropriately (see notes).
4. Prepare glass Pasteur pipets by fire-polishing their tips using a Bunsen burner (Figure 1). Prepare at least one pipet per individual culture, additional spares are recommended.

**Figure 1:**
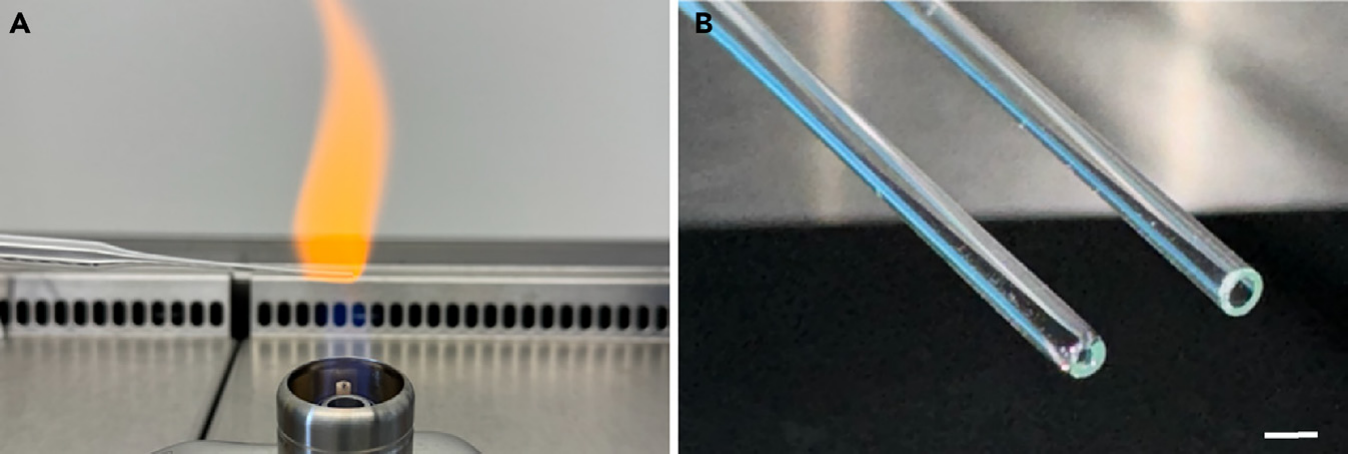
Preparation of glass Pasteur pipets (A) To fire-polish a glass Pasteur pipet, place the tip in the flame of a Bunsen burner for a couple of seconds while continuously rolling the pipet. (B) An example of a glass Pasteur pipet before (right) and after (left) fire-polishing. Scale bar = 1 mm.
5. Pre-cool centrifuges to 4°C.

***Alternatives:*** Culture matrices such as Matrigel or Collagen may be used in place of BME2 based on the specific requirements of the organoid culture (Step 24). However, variations in the physical properties of these matrices may require additional washes to ensure complete matrix removal and facilitate successful microtissue generation (Steps 35–37). To enhance the dissolution and effective removal of stiffer matrices (e.g., Collagen), extend the incubation time on ice following organoid harvesting.

**CRITICAL:** For successful formation of microtissues, it is essential to use round U-bottom plates with strong anti-adhesive properties.

***Alternatives:*** We have gained the best results with U-bottom plates provided by FaCellitate (see key resources table), however alternatives from other suppliers can be tested.

**CRITICAL:** To prevent attachment of liver progenitor organoids during their 15-day differentiation period, it is important to use untreated plates. In this case, pre-incubation of the plates at 37°C before seeding liver progenitor organoid pieces for differentiation is not needed.

## Key resources table

**Table:** 

REAGENT or RESOURCE	SOURCE	IDENTIFIER
**Antibodies**		

Anti-cleaved caspase-3 (Asp175) (1:200)	Cell Signaling Technology	Cat#9661S; RRID:AB_2341188
Donkey anti-rabbit, Alexa Fluor Plus 647 (1:500)	Thermo Fisher Scientific	Cat#A-31573; RRID:AB_2536183
Z-VAD-FMK (50 μM)	AbMole	Cat#M3143; CAS:187389-52-2
4′,6-diamidino-2-phenylindole dihydrochloride (DAPI) (1:2,000)	Toronto Research Chemicals	D416050; CAS:28718-90-3

**Chemicals, peptides, and recombinant proteins**		

Cultrex PathClear reduced growth factor basement membrane extract type 2	Bio-Techne	Cat#3533- 005-02
Collagenase A	Roche	Cat#10103586001
Dispase II	Sigma-Aldrich	Cat#102441796
DNase I	Sigma-Aldrich	Cat#DN25
TrypLE express enzyme (1X), phenol red	Gibco	Cat#2605010
DPBS, no calcium, no magnesium	Gibco	Cat#14190094
Advanced DMEM/F12	Thermo Fisher Scientific	Cat# 12634-010
HEPES	Thermo Fisher Scientific	Cat#15630-056
GlutaMAX	Thermo Fisher Scientific	Cat#35050-068
Penicillin/streptomycin	Thermo Fisher Scientific	Cat#15130-122
R-spondin1	prepared in-house	N/A
N21	R&D Systems	Cat#AR008
Nicotinamide	Sigma-Aldrich	Cat#N0636-100G
Noggin-Fc	IPA therapeutics	Cat#N002
N-acetyl-L-cysteine	Sigma-Aldrich	Cat#A9165
hFGF10	PeproTech	Cat#100-26
[Leu15]-gastrin I	Sigma-Aldrich	Cat#G9145-1MG
mEGF	PeproTech	Cat#315-09
hHGF	PeproTech	Cat#100-39
WNT surrogate-Fc	IPA therapeutics	Cat#N001
Y-27632	AbMole Bioscience	Cat#M1817
HepatiCult organoid basal medium (human)	STEMCELL Technologies	Cat#100-0387
HepatiCult organoid differentiation supplement (human)	STEMCELL Technologies	Cat#100-0388 https://cdn.stemcell.com/media/files/manual/10000008300-Initiation_Growth_and_Differentiation_of_Human_Hepati.pdf
Dexamethasone	Tokyo Chemical Industry	Cat#D1961
RapiClear 1.49	SunJin Lab	Cat#RC149001
Bovine serum albumin (BSA)	Capricorn Scientific	Cat#BSA-DG-100G
Triton X-100	Sigma-Aldrich	Cat#X100-500ML
Paraformaldehyde, 16% w/v aq. soln., methanol-free	Thermo Fisher Scientific	Cat#043368.9M
PBS tablets	Gibco	Cat#18912014

**Experimental models: Cell lines**		

Rosa26-CreERT2; mTmG liver progenitor organoids	Krotenberg Garcia et al.^1^	N/A
Villin-Cre^ERT2^*Apc*^fl/fl^*Kras*^G12D/WT^*Tr53*^fl/R172H^ cancer organoids	Fumagalli et al.^10^	N/A

**Software and algorithms**		

FlowJo 10.10.0	BD Biosciences	https://www.bdbiosciences.com/en-nl/products/software/flowjo-v10-software
Imaris 10.1.0	Oxford Instruments	https://imaris.oxinst.com/

**Other**		

Eppendorf centrifuge 5702 R	Eppendorf	Cat#5703KM622364
Eppendorf mini spin centrifuge	Eppendorf	Cat#5453KI907543
EVOS M5000 imaging system	Invitrogen	Cat#AMF5000
Confocal laser scanning microscope fast Airyscan	Carl Zeiss	LSM880
Plugged disposable glass Pasteur pipettes 150 mm	Volac	Cat#D810/PP
5 mL polypropylene round-bottom tube	Falcon	Cat#352053
5 mL polystyrene round-bottom tube with 35 μm cell-strainer cap	Falcon	Cat#352235
15 mL centrifuge tubes	Corning	Cat#430791
SafeSeal reaction tube, 1.5 mL, PP, PCR performance tested	Sarstedt	Cat#72.706.400
Multiwell plate for suspension culture, 12 well	Greiner Bio-One	Cat#665102
BIOFLOAT 96-well cell culture plate with ultra-low attachment surface	faCellitate	Cat#F202003
iSpacer 0.05 mm, double-sided sticky, 4 circular wells	SunJin Lab	Cat#IS204
Kimwipes	Kimtech Science	Cat#S7552

***Alternatives:*** Growth factors, such as mEGF and hHGF, may be replaced with equivalent products from alternative suppliers. Optimization is recommended and it is advisable to consult manufacturer's instructions when alternatives are used.

## Materials and equipment

### Culture media recipes

Adapted from Broutier et al.^12^

**Table:** Basic culture medium

Reagent	Stock concentration	Final concentration	Amount
Advanced DMEM/F-12	N/A	N/A	500 mL
HEPES	1 M	10 mM	5 mL
GlutaMAX	100X	1%	5 mL
Penicillin/Streptomycin	10.000 U/mL	100 U/mL	5 mL
**Total**	**–**	**–**	**515 mL**

Prepare in sterile environment, store at 4°C for up to 2 months.

**Table:** Digestion solution

Reagent	Stock concentration	Final concentration	Amount
Basic culture medium	N/A	N/A	10 mL
Dispase II	N/A	0.125 mg/mL	1.25 mg
Collagenase A	N/A	0.125 mg/mL	1.25 mg
DNase I	N/A	0.1 mg/mL	1 mg
**Total**	**–**	**–**	**10 mL**

Prepare fresh every time in sterile environment.

**Table:** Expansion medium

Reagent	Stock concentration	Final concentration	Amount
Basic culture medium	N/A	N/A	45.625 mL
R-spondin1	100%	5%	2.5 mL
N21	50x	1x	1 mL
Nicotinamide	1 M	10 mM	500 μL
Noggin-Fc	100%	0.5%	250 μL
N-acetyl-L-cysteine	500 mM	1 mM	100 μL
hFGF10	500 μg/mL	100 ng/mL	10 μL
[Leu15]-gastrin I	100 μg/mL	100 ng/mL	5 μL
mEGF	500 μg/mL	50 ng/mL	5 μL
hHGF	500 μg/mL	50 ng/mL	5 μL
**Total**	**–**	**–**	**50 mL**

Prepare in sterile environment, store at 4°C for up to 2 weeks.

**Table:** Complete HepatiCult Organoid Differentiation Medium (ODM)

Reagent	Stock concentration	Final concentration	Amount
HepatiCult Organoid Basal Medium (Human)	N/A	93.9%	46.95 mL
HepatiCult Organoid Differentiation Supplement (Human)	N/A	5%	2.5 mL
Penicillin/ Streptomycin	10.000 U/mL	100 U/mL	500 μL
Dexamethasone	3 mM	3 μM	50 μL
**Total**	**–**	**–**	**50 mL**

Prepare in sterile environment. Medium without dexamethasone can be stored at 4°C while protected from light for up to 2 weeks. Add Dexamethasone directly before use.

### Solution recipes

**Table:** Fixation solution

Reagent	Stock concentration	Final concentration	Amount
Paraformaldehyde	16%	8%	20 mL
PBS	2X from tablets	1X	20 mL
**Total**	**–**	**–**	**40 mL**

Store at −20°C for up to 1 month, avoid freeze-thaw cycles.

**CRITICAL:** Paraformaldehyde is hazardous; always wear chemical-resistant clothing and gloves to prevent skin contact, perform all steps involving paraformaldehyde in a chemical safety hood to minimize inhalation of vapors and dispose waste according to local regulations.

**Table:** Blocking solution

Reagent	Stock concentration	Final concentration	Amount
BSA	N/A	5%	2.5 g
TX-100	10%	0.2%	1 mL
PBS	1X from tablets	1X	49 mL
**Total**	**–**	**–**	**50 mL**

Store at 4°C for up to 1 month.

**Table:** Washing solution

Reagent	Stock concentration	Final concentration	Amount
TX-100	10%	0.1%	0.5 mL
PBS	1X from tablets	1X	49.5 mL
**Total**	**–**	**–**	**50 mL**

Store at room temperature (18°C–25°C) for up to 6 months.

**Table:** Dissociation solution

Reagent	Stock concentration	Final concentration	Amount
Basic culture medium	N/A	N/A	1 mL
Dispase II	N/A	5 mg/mL	5 mg
Collagenase A	N/A	5 mg/mL	5 mg
**Total**	**–**	**–**	**1 mL**

Prepare fresh every time in sterile environment.

**Table:** FACS buffer

Reagent	Stock concentration	Final concentration	Amount
PBS0	N/A	N/A	9.754 mL
N21	50X	1x	200 μL
N-acetyl-L-cysteine	500 mM	1.25 mM	25 μL
hEGF	0.5 mg/mL	50 ng/mL	1 μL
Y-27632	10 mM	10 μM	10 μL
DAPI	1 mg/mL	1 μg/mL	10 μL
**Total**	**–**	**–**	**10 mL**

Prepare in sterile environment, store at 4°C for up to 2 weeks.

## Step-by-step method details

### Generation and passaging of liver progenitor organoids

**Timing: 3–4 days**

The organoid lines used in the protocol have previously been established^1^^,^^10^^,^^13^ and therefore did not require institutional or local permissions. When isolation of new murine liver progenitor organoids lines is needed, the steps described in this section based on the protocol described by Broutier et al.^12^ can be followed and count an extra 1–2 weeks. Before starting, ensure local and institutional permissions to obtain or generate the organoids are in place. For a detailed explanation please refer to the original publication.

1. Prepare and prewarm the digestion solution to 37°C.
2. Place freshly isolated murine liver tissue in a 100-mm Petri dish and mince the tissue into small pieces (±0.5 mm^3^).
3. Transfer the minced tissue to a 50-mL tube with 10 mL of ice-cold Basic culture medium and allow tissue pieces to settle.
4. Discard the supernatant containing debris such as fat and red blood cells (±7.5 mL) and repeat washing once.
5. Remove remaining wash medium and add ∼10 mL of the digestion solution.
6. Incubate the digestion mixture on a shaker at 37°C for 45 min.
7. Check an aliquot of the solution for the presence of clean duct structures, if none are present replace the digestion solution and return to the shaker at 37°C.
8. Perform this check every 20–30 min until clean ducts are present (usually after ±2 h or when tissue pieces are no longer visible by naked eye).
9. Transfer the supernatant to a 15-mL tube and add ice-cold Basic culture medium to a volume of 15 mL.
10. Centrifuge at 300 x g for 5 min at 4°C to pellet the material.
11. Repeat wash once.
12. Enrich by hand-picking duct structures (refer to ref. ^12^ for detailed guidelines) or, when the material contains little contamination of other cell types or debris, plate part of the pellet directly in BME2 (2–3 different concentrations).
13. Invert the plate carefully and incubate at 37°C and 5% CO_2_ for 15–30 min to allow the BME2 to solidify.
14. Gently add 750 μL of room-temperature (18°C–25°C) Expansion medium per well.
15. Incubate at 37°C and 5% CO_2_.
16. Passage organoids every 3–4 days (see steps 17–27).

***Note:*** Organoids are delicate and maintaining them at low temperatures during passaging helps to preserve their functional integrity and viability. Therefore, it is recommended to use ice-cold reagents and maintain cells on ice throughout execution of the protocol.

17. Harvest organoids by scraping the bottom of the well while pipetting up and down with a p1000 pipet using ice-cold Basic culture medium. Ensure that all BME2 drops are detached and disrupted.
18. Collect the organoid suspension in 15 mL centrifuge tubes and keep on ice.

***Optional:*** For harvesting all organoid material, it is recommended to rinse the well with an additional 1 mL of ice-cold Basic medium and collect any remaining content.

19. Centrifuge at 300 x g at 4°C for 3 min. Three clear layers should be formed; medium, BME2 and the pellet of organoids (top-to-bottom, Figure 2).

**Figure 2:**
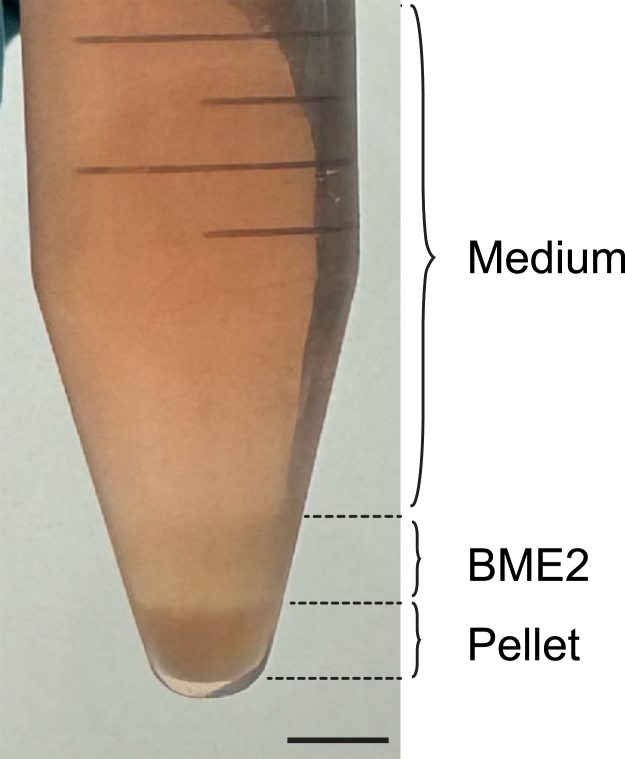
Liver progenitor organoids before breaking and seeding for differentiation Overview of the three layers formed by centrifugation of liver progenitor organoid suspension. Medium (top), BME2 (middle) and organoid pellet (bottom). Scale bar = 5 mm.
20. Remove the medium and BME2 layers carefully without disrupting the organoid pellet.
21. Resuspend the pellet in 1 mL of ice-cold Basic culture medium.
22. Use a fire-polished glass Pasteur pipet to mechanically disrupt the organoids.

**Figure 3:**
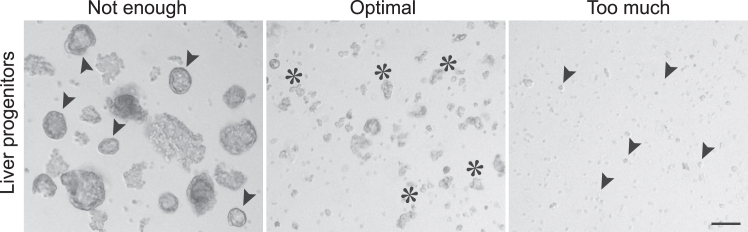
Generation of small fragments of liver progenitor organoids Bright-field examples of mechanically disrupted liver progenitor organoids that need to be further disrupted (left, indicated by arrowheads), have the optimal size for subsequent seeding and differentiation (middle, indicated by asterisks) and have been disrupted too much (right, indicated by arrowheads). Scale bar = 100 μm.

**CRITICAL:** Pipet up and down 10-20 times while pressing the pipet tip against the bottom of the tube until small fragments are formed (Figure 3).

***Note:*** Immediately before use, sterilize glass Pasteur pipets by briefly passing them through a flame. Pre-wet the pipet with Basic culture medium and only aspirate a small volume at a time to avoid attachment of the organoids to the glass surface.

23. Centrifuge suspension to pellet at 300 x g at 4°C for 3 min.
24. Carefully aspirate supernatant and resuspend the pellet in BME2 and Basic culture medium with a ratio of 2:1. Ensure the mixture is homogenous before seeding.
25. Evenly dispense the BME2/organoid mixture in 10–12 μL droplets in a 12-well suspension plate.
26. Invert the plate carefully and incubate at 37°C and 5% CO_2_ for 15–30 min to allow the BME2 to solidify.
27. Gently add 750 μL of room-temperature (18°C–25°C) Expansion medium per well. Incubate at 37°C and 5% CO_2_.

### Differentiation of liver progenitor organoids

**Timing: 15 days**

This section outlines the essential steps for preparation and subsequent differentiation of murine liver progenitor organoids. The 15-day differentiation protocol described below is based on the protocol developed by STEMCELL Technologies (see key resources table for link) with some adaptations for the successful generation of mixed microtissues.

**CRITICAL:** Passage liver progenitor organoids three days prior to the start of differentiation to allow sufficient growth and ensure they can be effectively fragmented into small pieces before initiating the differentiation process. Calculate the input material needed for the desired number of microtissues (Table 1).

***Note:*** For pure microtissues, only use the input for one of the populations.

**CRITICAL:** Depending on the pellet size (actual ratio) of wild-type and cancer cells after harvesting, adjustments may be necessary to achieve the desired ratio of wild-type and cancer cells for microtissue formation, as shown in Table 2.

28. Passage liver progenitor organoids by following steps 17–27 of the protocol.
29. After plating and addition of Expansion medium (step 27), incubate at 37°C and 5% CO_2_ for 5 days, with a medium refreshment on day 3.
30. On day 5, switch liver organoid cultures to complete HepatiCult Organoid Differentiation Medium (ODM).
31. Refresh with 750 μL fresh complete HepatiCult ODM per well on days 8 and 11.

**Figure 4:**
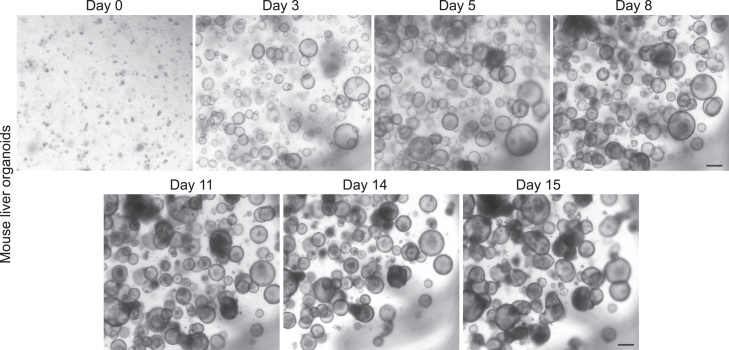
Overview of the 15-day differentiation of liver progenitor organoids Bright-field images depicting the morphology of liver progenitor organoids on medium refreshment days during the 15-day differentiation protocol. Scale bar = 200 μm.

**CRITICAL:** Optimal breaking of the organoids is essential. Therefore, it is recommended to monitor the progress using a light-microscope after every 5-10 times of pipetting. Stop the breaking of the organoids before they become single cells (Figure 3).

**CRITICAL:** The seeding density is an important determinant of the differentiation protocol. Overcrowding results in loss of material by plate attachment and reduces the differentiation efficiency, while low densities do not yield sufficient material. Refer to Figure 4 for the preferred density.

***Note:*** The matrix drops are easily disrupted during media refreshment, careful aspiration and addition of medium is recommended.

**CRITICAL:** It is important to aliquot and supplement the required volume of HepatiCult ODM with 3 mM dexamethasone (‘complete HepatiCult ODM’) on each day that a full-medium change is required. Equilibrate at 18°C–25°C before each use. (Complete) HepatiCult ODM is light-sensitive; therefore, minimize exposure to light by switching of the light in the hood during preparation and medium refreshment. For detailed information about medium composition, refer to the protocol manual.

***Note:*** For organoid cultures that require passaging every 2–3 days, such as cancer organoids, ensure to passage them 2–3 days prior to microtissue generation (i.e., on days 12-13 of liver differentiation). Calculate the input material based on the desired number and mixing-ratio of microtissues (Table 1).

32. On day 14, refresh both liver and cancer organoid cultures with fresh complete HepatiCult ODM supplemented with Noggin to maintain both populations in optimal conditions.

**CRITICAL:** Determining the optimal medium conditions to maintain organoid cultures in a functional and potentially fully differentiated state is essential. When mixing differentiated liver cells with other cell populations, it is recommended to perform medium screening to identify the best culture conditions for both populations. For instance, Noggin is added in this protocol to support *Apc*^−/−^*Kras*^G12D/WT^*Trp53*^-/R172H^ intestinal cancer organoids Refer to the troubleshooting section for examples and detailed guidance.

33. On day 15, the differentiated liver and cancer organoids that were cultured in complete HepatiCult ODM supplemented with Noggin are ready for generation of microtissues.

***Note:*** It is recommended to monitor the progress of liver organoids during the 15-day differentiation by acquiring images every 2–3 days (Figure 4).

**Table 1:** Recommended input material for the generation of one microtissue

	Ratio 1:1	Ratio 2:1	Ratio 3:1	Ratio 4:1
Wild-type	1/2 × 12w	1/2 × 12w	1/2 × 12w	1/2 × 12w
Cancer	1/2 × 12w	1/4 × 12W	1/6 × 12W	1/8 × 12W

**Table 2:** Recommended volumes for generation of microtissues with a 2:1 mixing ratio of wild-type (WT) and cancer cells

Pellet size (ratio)		Resuspension volume (μL)		Mixing volume (μL)		No. of microtissues		
WT	Cancer	WT	Cancer	WT	Cancer	Mixed	Pure WT	Pure cancer
2	1	200 μL	200 μL	100 μL	100 μL	1	1	1
1	1	200 μL	200 μL	100 μL	50 μL	1	1	3
3	1	200 μL	200 μL	67 μL	100 μL	1	2	1
4	1	200 μL	200 μL	50 μL	100 μL	1	3	1

Use the indicated volumes to compensate for discrepancies in the pellet size of the two cell populations.

### Generation of microtissues

**Timing: 3 days**

This section outlines a series of steps required for generating microtissues using intestinal *Apc*^−/−^*Kras*^G12D/WT^*Trp53*^-/R172H^ cancer and differentiated liver progenitor organoids, referred to as hepatocyte-like organoids.

34. Harvest hepatocyte-like and cancer organoids by following steps 17–20 of the protocol.
35. After removal of the medium and BME2 layers (step 20), add 5 mL of ice-cold Basic culture medium to the pellet and resuspend using a 10 mL serological pipet.
36. Centrifuge at 300 x g at 4°C for 3 min. The BME2 layer should be significantly reduced (Figure 5).

**Figure 5:**
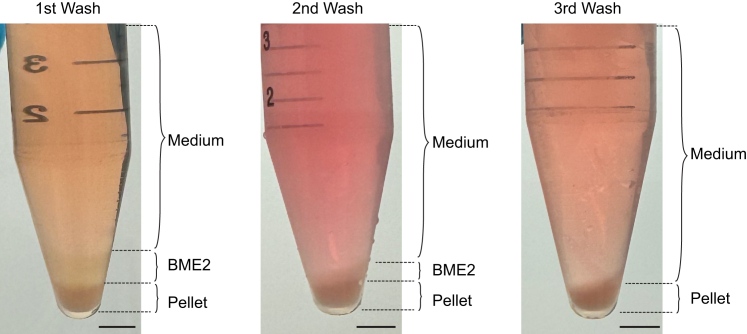
Preparation of hepatocyte-like organoids for microtissue generation Overview of the three layers formed by centrifugation after each of the three washing steps before breaking hepatocyte-like organoids. After the third washing step, BME2 is significantly reduced. Medium (top), BME2 (middle) and organoid pellet (bottom). Scale bar = 5 mm.
37. Repeat steps 35 and 36 twice.

**CRITICAL:** For successful microtissue generation, ensure there are no BME2 leftovers in the tube before proceeding in the next step of breaking organoids into pieces (Figure 5).

38. Remove the supernatant carefully without disrupting the organoid pellet.
39. Resuspend the pellet in 1 mL of ice-cold Basic culture medium.
40. Use a fire-polished glass Pasteur pipet to mechanically disrupt both cancer and hepatocyte-like organoids individually.

**Figure 6:**
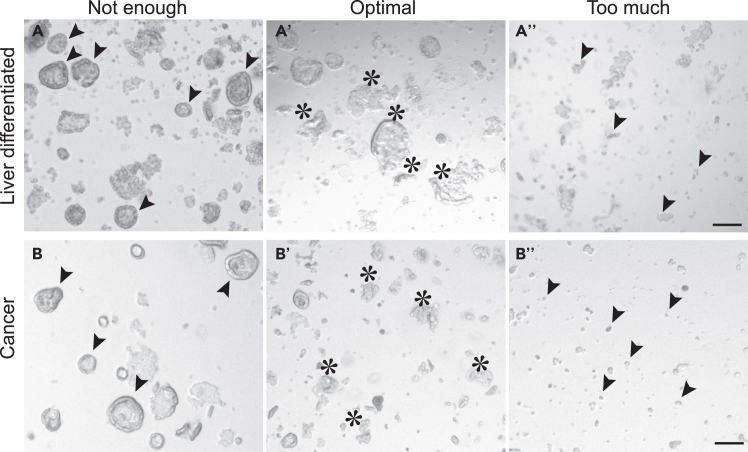
Generation of fragments of hepatocyte-like and cancer organoids for microtissue formation (A and B) Bright-field images of mechanically disrupted hepatocyte-like (A) and cancer (B) organoids that need to be further disrupted (A, B, indicated by arrowheads), have the optimal size for microtissue formation (A′, B′, indicated by asterisks) and have been disrupted too much (A″, B″, indicated by arrowheads). Scale bar = 100 μm.

***Note:*** For liver organoids, gently pipet up and down 5-10 times until medium-sized fragments are formed. Subsequently, disrupt cancer organoids vigorously into smaller fragments by pressing the pipet tip against the bottom of the tube (Figure 6).

**CRITICAL:** Avoid breaking hepatocyte-like cells into very small fragments or single cells, as this can induce an injury response and compromise the differentiation state of the cells. Monitor the progress using a light-microscope after every 5-10 times of pipetting (Figure 6).

41. Centrifuge at 300 x g at 4°C for 3 min.
42. Prepare one Eppendorf tube for each of the conditions (Pure wild-type, Pure cancer and Mixed wild-type: cancer) and add the corresponding volumes of each population to generate microtissues with the desired mixing ratio (Table 2).

**CRITICAL:** Generating mixed microtissues of wild-type and cancer cells in a 2:1 ratio depends on the input material. Therefore, it is essential to compare the pellets of both populations, resuspend them, preferably, in the same volume of Basic culture medium and add the appropriate volume of cell suspension for each condition (Table 2).

43. Centrifuge at 300 x g at 4°C for 3 min.
44. Carefully discard the supernatant and add approximately 20–25 μL of HepatiCult ODM supplemented with Noggin.
45. Incubate the Eppendorf tubes at 37°C for 30 min while protected from light to facilitate cell aggregation and microtissue formation.

**CRITICAL:** Resuspension of the pellet in a small volume before incubation is important for efficient cell aggregation and formation of mixed microtissues.

46. After incubation, for each desired microtissue add an additional 100 μL HepatiCult ODM supplemented with Noggin to the aggregates.
47. Carefully resuspend the aggregate suspension and distribute 100 μL per well in a 96-well U-bottom plate to form one microtissue per well.
48. Incubate at 37°C and 5% CO_2_ for at least 3 days to allow formation. After this period, microtissues can be used for further analysis as described in the following sections.

**Figure 7:**
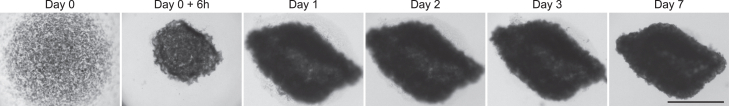
Aggregation and formation of microtissues Bright-field images of cell aggregate suspension in 96-well U-bottom plates (left) and microtissue formation over time. Scale bar = 1 mm.

**Figure 8:**
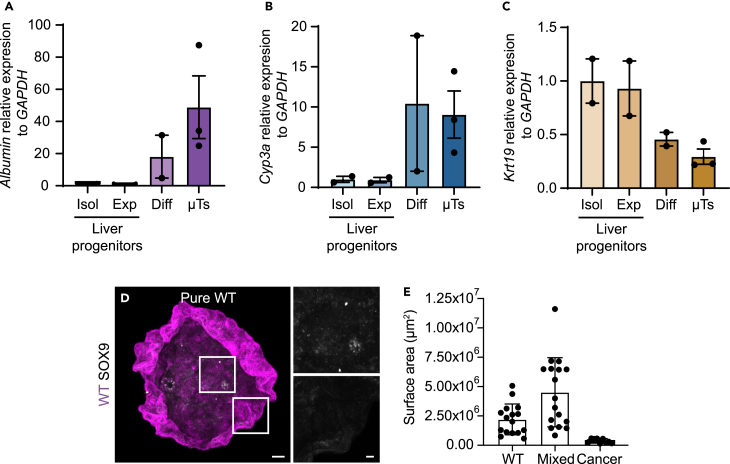
Quality control for input material and microtissues (A–C) Graphs displaying the expression of hepatocyte marker genes *Albumin* (A) and *Cyp3a* (B) and liver progenitor marker gene *Krt19* (C) relative to the housekeeping gene *GAPDH*, in liver progenitor organoids cultured for 3 days in isolation and expansion medium, in liver progenitor organoids at day 15 of the differentiation protocol and in microtissues 3 days after plating (Liver progenitors and Diff *n* = 2 and μTs *n* = 3, ± SD). (D) Representative 3D-reconstructed stitched confocal images of a pure WT microtissue stained for SOX9 (gray); nuclei are visualized with DAPI (blue). The insets display a 2.5x magnification of the area in the white box. (E) Quantification of the surface area of pure and mixed microtissues; each dot represents one microtissue (WT *n* = 16, Cancer *n* = 9 and Mixed *n* = 17 microtissues, ± SD). Scale bars represent 100 μm (stitched image) and 10 μm (magnification). Figures (A)–(D) are reprinted with permission from Krotenberg Garcia et al.^1^

***Note:*** It is recommended to confirm the equal distribution of aggregates using a light-microscope and monitor the progression of microtissue formation daily using a light-microscope (Figure 7).

**CRITICAL:** Ensure successful microtissue generation by resuspending them in their medium on day 3. If they remain intact, they are ready for use. Furthermore, quality of the generated microtissues depends on the maintenance of hepatocyte-like cells in a differentiated state. This can be assessed by measuring the expression levels of differentiation markers (Figures 8A–8C) or performing immunostainings for detecting the absence of progenitor markers (Figure 8D).

***Note:*** When using input material that is of sufficient quality as described in the previous steps (e.g. well-differentiated hepatocyte-like cells, well-maintained viable cancer cells and optimal density) the success rate of microtissue formation is 90–95%. The size of generated microtissues is dependent on the cell type of origin, ranging from ±0.4 mm^2^ for pure cancer to ±4.5 mm^2^ for mixed microtissues (Figure 8E).

### Immunofluorescence staining and mounting of microtissues

**Timing: 3–4 days**

This section describes how to perform immuno-fluorescence staining and mounting of mixed microtissues. The indicated volumes are optimized for individual wells in a 96-well U-bottom plate.

49. To fix microtissues, carefully remove 50 μL of the medium and add 50 μL of Fixation solution (see “materials and equipment” section) to the remaining 50 μL of medium in each microtissue-containing well.

**CRITICAL:** Ensure to not touch the microtissue during this step.

50. Incubate for 30–35 min at 18°C–25°C while protected from light.

**CRITICAL:** At the end of the incubation time, confirm successful fixation and microtissue integrity by carefully aspirating a bit of the medium and moving the microtissue in the well. The structure should be coherent and intact. Discard broken or disrupted microtissues.

51. Remove fixation solution and wash twice with 100 μL of PBS0.

**Pause point:** After fixation, microtissues can be stored in PBS0 for 2–3 weeks at 4°C, protected from light, before continuing with the staining protocol.

52. Carefully aspirate PBS0, then add 75 μL of Blocking solution (see “materials and equipment” section) and incubate at 4°C for 8–16 h while protected from light, to block and permeabilize microtissues.
53. Remove Blocking solution and add 75 μL of the desired primary antibodies diluted in Blocking solution. Protect from light and incubate at 4°C for 12–16 h.
54. Wash three times with 150 μL of Washing solution (see “materials and equipment” section) at 18–25°C for at least 5 min each, these steps can be extended.
55. Add 75 μL of the appropriate secondary antibodies diluted in Blocking solution. Protect from light and incubate at 4°C for 12–16 h.

**Pause point:** The block/permeabilization and primary and/or secondary antibody incubation steps can all be extended for 2–3 days while protected from light at 4°C.

***Note:*** Dye solutions, e.g., DAPI or Phalloidin, can be included during the secondary antibody incubation (step 55).

**CRITICAL:** To avoid crosstalk, selection of secondary antibodies and dyes should be made by excluding fluorophores with overlapping spectra to those that are already expressed in microtissues.

56. Wash twice with 150 μL of Washing Buffer at 18–25°C for 5 min each.
57. Add 150 μL of PBS0 after the last washing step.

**CRITICAL:** During all washing steps, exchange liquids with great care and ensure that microtissues are not accidently aspirated.

**Pause point:** After this step, stained microtissues can be stored long-term at 4°C, protected from light. To avoid signal loss, it is recommended to proceed with imaging within one week. Add an extra volume of PBS0 and/or seal the plate with parafilm to prevent the microtissues from drying out.

**Figure 9:**
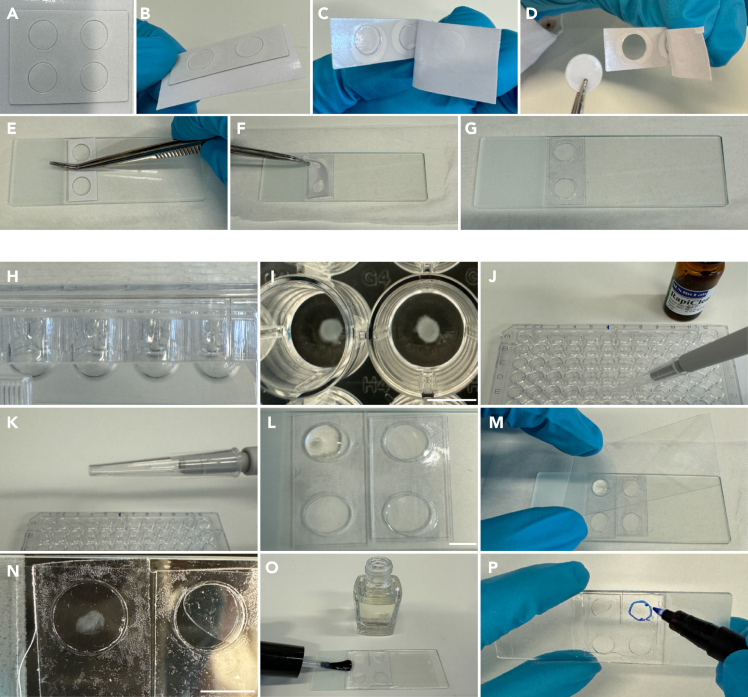
Steps for mounting microtissues for imaging analysis (A–D) Peel off the protective liner from one side and remove the circular well inserts from an iSpacer pair, (E–G) Apply the iSpacer pair vertically to the slide and remove the second protective liner, (H and I) Overview of U-bottom 96-well plate used for microtissue formation, (J–L) Resuspend the microtissue in RapiClear solution and use a pre-cut P200 to transfer it to the iSpacer confinement, (M–P) Place a coverslip on top of the iSpacers, seal by applying nail polish around the edges of the coverslip and mark the microtissue to easily detect it during imaging. Scale bar = 5 mm.

58. To mount microtissues on a microscope slide (25 × 65 mm), label slides and clean the surface of the slide using Kimwipes.
59. Use thin (0.05 mm) adhesive double-sided spacers (iSpacers, Figure 9A) to create confinements on the slide for each to-be-mounted microtissue:
  a. Cut the required number of confinements from an iSpacer (Figure 9B).
  b. Peel off the protective liner from one side of the iSpacers (Figure 9C).
  c. Carefully remove the circular well inserts (Figure 9D).
  d. Vertically to the slide, apply 3–4 pairs of iSpacers in a row on the dry slide surface, ensuring the sticky side adheres to the slide (Figure 9E).
  e. Gently press to seal the spacers onto the slide.
  f. Carefully remove the second protective liner from the iSpacers (Figure 9F).

**CRITICAL:** Verify that the iSpacers are fully adhered to the slide.

60. Aspirate PBS0 and use a pre-cut P200 pipet tip to add 15 μL of RapiClear clearing solution to each microtissue-containing well (Figure 9J).
61. Use the same pre-cut P200 pipet tip to immediately transfer each microtissue, suspended in 15 μL of RapiClear clearing solution, to a separate iSpacer confinement (Figure 9K).
62. Add an extra drop of RapiClear clearing solution on top of each microtissue using a new non-cut P200 pipet tip and place it centrally within the confinement (Figure 9L).
63. Quickly repeat steps 60–62 for all to be mounted microtissues.
64. Place a coverslip (24 × 50 mm, thickness No. 1.5) on top of the iSpacers to seal the microtissue-containing confinements (Figure 9M).
65. Gently press the edges of the coverslip to ensure proper sealing of the wells.
66. Carefully remove any excess solution from the coverslips using Kimwipes.
67. Apply clear nail polish around the edges of the coverslip to seal and ensure it remains immobilized (Figure 9O).
68. Circle the position of each microtissue on the microscope slide side to facilitate sample detection during imaging (Figure 9P).

**CRITICAL:** Mount quickly after the addition of RapiClear (step 60) as the solution will clear the microtissues and their transparency interferes with positioning (step 62) and marking (step 68) of the microtissues.

**Pause point:** After this step, mounted microtissue slides can be store in a light-protective case at 4°C for 2–3 days.

**Figure 10:**
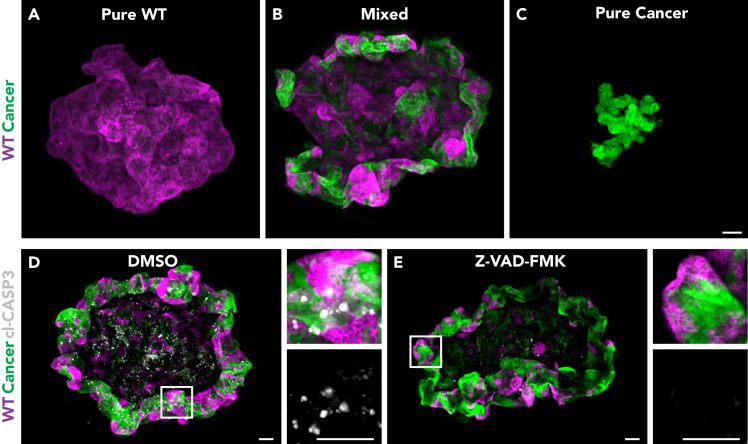
Imaging of cellular interactions in microtissues (A–C) Representative 3D-reconstructed stitched confocal images of pure WT (A), mixed (B), and pure cancer (C) microtissues. (D and E) Representative 3D-reconstructed stitched confocal image of a control (D) and Z-VAD-FMK treated mixed microtissue stained for cleaved caspase-3 (gray). The insets display a 3x magnification of the area in the white box. Scale bars = 100 μm.

***Note:*** To avoid loss of information, it is recommended to acquire Z-stacks covering the total thickness of each mixed microtissue (100–300 μm). The thickness of Z-slices should be determined based on the detail of information that is needed for further analysis. For example, a thickness of 5 μm is sufficient for analysis at a cellular resolution. The dimensions of a mixed microtissue exceed the size of an individual field of view, therefore using tiling (e.g., 6 × 6) to capture the entire area of a microtissue. After stitching the individual tiles, data can be further analyzed via 3D reconstruction or Z-projection.

69. Proceed to imaging of microtissues using a platform of choice. A confocal based platform is recommended, using a high NA 20x-25x Dry objective.
70. Acquire Z-stack and use tiles to capture the entire microtissue (Figure 10).

### Sorting cells from mixed microtissues

**Timing: 1 day**

This section describes the steps required for dissociating microtissues and subsequent sorting of viable individual cell populations.

71. Use pre-cut P200 pipet tips to transfer 4–5 microtissues to a 15 mL tube containing 1 mL of ice-cold Basic culture medium.
72. Centrifuge at 300 x g at 4°C for 3 min.
73. Carefully aspirate supernatant.
74. Add 1 mL of Dissociation solution (see “materials and equipment” section) to the microtissue pellet.

***Note:*** Initially, use a P200 pipet tip to break microtissues into smaller pieces, followed by mechanical disruption using a fire-polished glass Pasteur pipet by passing it through the pipet 10-15 times.

75. Incubate at 37°C for 2 min.
76. Use the same Pasteur pipet to further disrupt until small cell fragments are devoid of extracellular matrix.
77. Inactivate enzymatic activity by diluting the mixture with 9 mL of ice-cold Basic culture medium. Keep the tube on ice.

***Note:*** The protease activity of Dispase II and Collagenase A facilitates the dissociation of cells from their extracellular matrix, enabling the generation of small-cell clusters while preserving cell integrity and viability.

78. Centrifuge at 300 x g at 4°C for 3 min.
79. Carefully aspirate supernatant without disrupting the cell pellet.
80. Add 1 mL of TrypLE pre-warmed to 37°C to the pellet and resuspend it thoroughly using a fire-polished glass Pasteur by pipetting up and down 10 times.
81. Incubate at 37°C for 1 min.
82. Continue pipetting until single cells are formed.

**CRITICAL:** It is crucial to resuspend the cells once or twice during incubation with the Dissociation solution or TrypLE to avoid clump formation.

***Note:*** For dissociation of microtissues into single cells, TrypLE is recommended. Unlike alternative dissociation reagents, TrypLE is inactivated by dilution rather than by trypsin inhibitors. Refer to the supplier's guidelines for detailed instructions when using other dissociation reagents.

**Figure 11:**
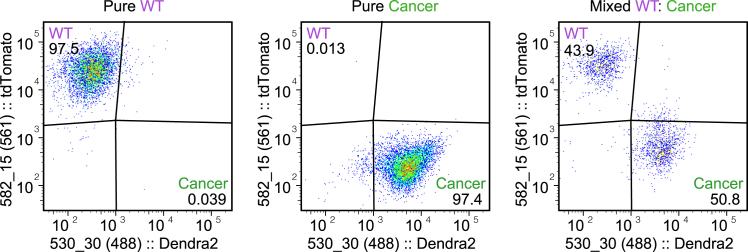
Analysis of cellular interactions in microtissues using cell sorting Scatter plots displaying flow cytometry sorting of viable wild-type (WT) and cancer cells from dissociated pure and mixed microtissues, the numbers in the corners display the percentage of sorted cells.

***Note:*** Before sorting, it is recommended to use diluted live/death-dyes of choice (e.g., DAPI, Thiazole red) to identify and sort viable cells of the desired populations. The number of viable single cells that can be typically retrieved from one microtissue is approximately 500-2000.

83. Inactivate TrypLE by diluting with 9 mL of ice-cold Basic culture medium while keeping on ice.
84. Centrifuge at 300 x g at 4°C for 3 min.
85. Carefully aspirate supernatant without disrupting the cell pellet and keep on ice.
86. Pre-wet the cell strainer cap of a 5 mL Round Bottom Polystyrene tube with 100 μL of ice-cold FACS buffer.
87. Add 250 μL of ice-cold FACS buffer and pass the single-cell pellet through the 35 μm cell strainer.
88. Keep cells on ice and immediately proceed to sorting using a platform of choice (Figure 11).

## Expected outcomes

This protocol describes the process of generating mixed microtissues to investigate the nature and outcome of competitive cellular interactions between intestinal *Apc*^−/−^*Kras*^G12D/WT^*Trp53*^-/R172H^ cancer and hepatocyte-like cells. Prior to microtissue formation, the differentiation state of hepatocyte-like organoids should be monitored throughout the 15-day protocol using bright-field microscopy (Figure 4). To further assess the differentiation of liver organoids, it is recommended to measure the expression levels of specific marker genes, such as *Albumin*, *Cyp3A* and *Krt19* (Figures 8A–8C).^1^^,^^12^ Using mixed microtissues to mimic liver metastasis, we previously showed that cancer cells exhibit a proliferative advantage over surrounding wild-type liver cells, with the latter serving as a scaffold for metastatic outgrowth (Figure 10A–10C and ref. ^1^).

Microtissues are technically related to other 3D culture systems, such as organoids and assembloids. The primary difference between these models is the degree of tissue complexity. Organoids are 3D miniature versions of the tissues from which they originate and are cultured in a supplied matrix.^16^ Assembloids are 3D self-organizing models created by fusing multiple organoids to study inter- and intra-tissue interactions, such as vascularized brain, neuro-mesodermal, and brain-cortex-spinal cord assembloids.^17^^,^^18^^,^^19^ The generation of assembloids depends on advanced co-culture protocols that enable the fusion and function of distinct regions within the model. Microtissues resemble assembloids in structure but are a simplified version as these 3D cell aggregates only contain cell types of a similar origin (e.g., epithelial cells) in a matrix-free environment.

Mixed microtissues can be leveraged to investigate the molecular mechanisms underlying competitive interactions in liver metastasis. Upon formation of mixed microtissues, the phenotypical features of each individual cell population should be closely examined. Immuno-fluorescence staining for specific markers can help establish a correlation between competition and pathway activation. For example, through staining for cleaved caspase-3 we previously demonstrated increased cell death of wild-type cells at the interface with cancer cells, highlighting the importance of short-range interactions in metastatic outgrowth (Figures 10 D and 10E and ref. ^1^).

Novel insight into specific pathways that have an instrumental role in the elimination of wild-type or the survival of cancer cell populations can be extracted by performing omics-approaches. For instance, using 3D mixed organoids to study the effect of cell competition in primary intestinal cancer, bulk RNA-sequencing revealed that wild-type intestinal cells are outcompeted by cancer cells by upregulating a fetal-like signature in a JNK-dependent manner.^13^ To this aim, sorting of viable cells of the two individual cell populations from mixed microtissues is essential to proceed with either RNA sequencing or proteomic analyses and identify gene trajectories orchestrating these processes (Figure 11). Importantly, the involvement of specific molecular pathways can be experimentally investigated using chemical or genetic manipulation. This also highlights the broad application of our model, as liver metastasis microtissues can be adapted to a screening platform for therapeutics and, it is easily adaptable to other liver-metastasizing cancers, such as breast and pancreatic cancer.

## Limitations

For generating liver metastasis microtissues, we made use of two murine adult stem cell-derived organoid lines. These organoids are only composed of epithelial cell types, allowing us to specifically investigate competitive interactions developed exclusively between wild-type liver and intestinal cancer epithelial cells. This approach excludes the potential impact of other (non-) cellular components that are typically present in the *in vivo* liver microenvironment. Therefore, the microtissues as described here, are less suitable for modeling metastases that exhibit growth patterns with high densities of stromal and immune cells, such as the desmoplastic growth pattern.^15^ The integration of other cell types into this model poses certain limitations, particularly concerning the media conditions required to support each cell population without inducing biased competition. Like the optimized media conditions required for generating microtissues from differentiated liver and intestinal cancer cells (step 32), further optimization of media recipes is needed to successfully generate different types of microtissues while maintaining the desired differentiation state of the involved cell types.

To assess the impact of competition in our model, we perform fixation and imaging analysis of microtissues three days post-formation. Handling microtissues before this time point can be complicated and its success depends on the strength of the cellular interactions and overall structural integrity. Furthermore, following dynamic interactions of individual cells within microtissues using time-lapse microscopy is crucial to gain insight into the cellular mechanisms that are at play during competition in liver metastasis. However, unlike 3D mixed organoids, that are grown in a matrix and thereby stabilized during long-term imaging,^20^ microtissues are devoid of any matrix and free-floating which is challenging for time-lapse microscopy. Therefore, further optimization is needed to immobilize these structures within a specific field of view over time. A potential solution involves spatially confining or embedding the microtissues in low concentrations of agarose or methylcellulose, a technique that has been successfully employed for the live imaging of zebrafish embryos over a period of three days.^21^

## Troubleshooting

### Problem 1

The complete HepatiCult ODM used for differentiating liver progenitor organoids may not support the optimal growth of other organoid lines intended for the generation of different mixed microtissues (step 32).

### Potential solution

To minimize biased competition within mixed microtissues, perform a medium screening using the complete HepatiCult ODM and make necessary adjustments to identify optimal conditions for the growth and maintenance of the differentiated states of the individual cell populations. For instance, to generate mixed microtissues between hepatocyte-like and cancer cells, the HepatiCult ODM was supplemented with Noggin, which is indispensable for cancer organoid growth. If this approach is ineffective, consider using and/or adjusting the liver differentiation medium described by Broutier et al. (ref. ^12^) and determine a common medium composition that supports the viability and differentiation state of the cell populations involved. As example, we optimized the co-culture media for the generation of mixed microtissues from hepatocyte-like and *Apc*^*−/−*^ small intestinal cells,^1^ as the latter showed a growth delay and increased death in the complete HepatiCult ODM supplemented with mEGF, Noggin and R-spondin1. After testing media with different compositions, we found that the liver differentiation medium as outlined by Broutier et al. (ref. ^12^), supplemented with Noggin, R-spondin1 and excluding DAPT and dexamethasone (*Apc* microtissue medium^1^) supported growth of *Apc*^*−/−*^ small intestinal organoids. Importantly, *Apc* microtissue medium was also compatible in maintaining the viability of hepatocyte-like cells.

### Problem 2

Differentiating organoids attach to the bottom of the plate (steps 28–33).

### Potential solution

Use uncoated multi-well plates for suspension culture for the differentiation protocol and prevent overcrowding of the cultures (Figure 4).

### Problem 3

Breaking hepatocyte-like cells into very small fragments or single cells can induce an injury response coupled and trans-differentiation to a progenitor state (step 40).

### Potential solution

Prevent harsh dissociation by gentle disruption hepatocyte-like organoids. Frequently monitor the process (every 2-3 times of pipetting up and down) by inspecting their morphology using a light-microscope until they reach the desired size (Figure 6).

### Problem 4

The cell pellet reduces in size during removal of the matrix (steps 35–37).

### Potential solution

To prevent disruption of the pellet, aspirate the medium and matrix using a P1000 instead of a vacuum-based aspiration system. Do not discard the medium when the pellet is accidentally disrupted, instead keep the medium and centrifuge again before continuation.

### Problem 5

The wells are empty after immuno-fluorescence staining protocol (step 57).

### Potential solution

Microtissues are easily aspirated during washes. Therefore, carefully remove liquids using a P200 pipet without touching the microtissue. Frequently monitor the plate to confirm that each well contains a microtissue.

### Problem 6

Microtissues stick to the wall of pre-cut P200 tips while transferring them on the microscope slide (step 61).

### Potential solution

Aspirate a small volume of PBS0 using this pre-cut P200 tip and try to release the suspended microtissue in PBS0 into a U-bottom well and repeat its mounting on the microscope slide.

### Problem 7

The mounted microtissues dry out and the fluorescent signal is lost over time (steps 69 and 70).

### Potential solution

Ensure that sticky side of the iSpacers is fully adhered to the microscope slide by gently pressing until no air bubbles are visible between the iSpacer and the glass slide (step 59). Make sure that all edges of the mounted slide are completely sealed with nail polish (step 67). If air bubbles do remain, image slides immediately to prevent signal loss (step 69).

### Problem 8

Sample navigation during imaging of cleared microtissues is difficult (step 69).

### Potential solution

Ensure rapid mounting of microtissues after addition of the RapiClear solution as this enhances their transparency (steps 60 and 61). In addition, place the microtissues in the center of the iSpacer confinements and mark their positions (steps 62 and 68, see also Figure 10).

### Problem 9

Clump formation and reduced cell viability during cell dissociation (steps 74, 76, 80 and 82).

### Potential solution

Avoid prolonged incubation and/or too little resuspension of microtissues during incubation with the Dissociation solution or TrypLE. To avoid loss of viable cells, manually remove cell debris by gently swirling with a glass Pasteur pipet. Alternatively, add DNase to the incubation mixture to prevent clump formation by degrading DNA released from lysed cells.

## Resource availability

### Lead contact

Further information and requests for resources and reagents should be directed to and will be fulfilled by the lead contact, Saskia J.E. Suijkerbuijk (s.j.e.suijkerbuijk@uu.nl).

### Technical contact

Technical questions on executing this protocol should be directed to and will be answered by the technical contact, Maria Lamprou (m.lamprou@uu.nl).

### Materials availability

This study did not generate new unique reagents.

### Data and code availability

This study did not generate new data or code.

## Acknowledgments

We thank Toni van Capel and the Flow Cytometry facility of Utrecht University for technical support with cell sorting, Ilya Grigoriev and the Biology Imaging Center of Utrecht University for technical support with imaging, and members of the Division of Developmental Biology for input and technical support. This work was financially supported by Dutch Cancer Society Young Investigator Grant 11491/2018-1 (to S.J.E.S.).

## Author contributions

M.L.: methodology, investigation, validation, writing – original draft, and visualization. A.K.G.: conceptualization, methodology, investigation, and writing – review and editing. S.J.E.S.: conceptualization, methodology, writing – original draft, writing – review and editing, supervision, and funding acquisition.

## Declaration of interests

The authors declare no competing interests.
